# Comparison of Frailty and Autonomy Levels in Older Adults According to Place of Residence

**DOI:** 10.3390/healthcare14060721

**Published:** 2026-03-12

**Authors:** Andrea Machado, Pedro Forte, Filipe Rodrigues

**Affiliations:** 1 ESECS—Polytechnic of Leiria, 2411-901 Leiria, Portugal; 2023113400@my.ipleiria.pt; 2 Department of Sports Sciences, Instituto Politécnico de Bragança, 5300-253 Bragança, Portugal; pedromiguel.forte@iscedouro.pt; 3 Research Centre for Active Living and Wellbeing (LiveWell), Instituto Politécnico de Bragança, 5300-253 Bragança, Portugal; 4 Department of Sports, Higher Institute of Educational Sciences of the Douro, 4560-708 Penafiel, Portugal; 5 Research Center in Sport, Health and Human Development (CIDESD), 5000-558 Vila Real, Portugal

**Keywords:** ageing, autonomy, frailty, older adults, place of residence

## Abstract

**Background/Objectives**: The present study aimed to compare levels of frailty and functional independence in older adults according to their place of residence and to analyze the associations between age, frailty, and autonomy, considering sex. **Methods**: A cross-sectional analytical study was conducted with a sample of 77 older adults (80.16 ± 8.68 years), divided into community-dwelling (*n* = 38) and institutionalized (*n* = 39) participants. Data collection included sociodemographic characterization and the assessment of frailty and autonomy. **Results**: Older adults in the community showed significantly higher levels of autonomy (94.21 ± 10.81) compared to those who were institutionalized (75.53 ± 23.21; *p* < 0.001). No significant differences were found in frailty levels between the groups (*p* = 0.674). Correlation analysis revealed a strong negative association between frailty and autonomy, which was more pronounced in institutionalized older adults (r = −0.64) and in males (r = −0.72). Age was only correlated with the loss of autonomy in men (r = −0.43). **Conclusions**: Place of residence is a critical determinant of functional autonomy but not of perceived frailty. The results highlight a health-survival paradox, where men exhibit a more abrupt functional decline associated with frailty. The implementation of gerontomotricity programs in residential care facilities is suggested to mitigate learned dependency.

## 1. Introduction

Population aging is one of the most significant demographic phenomena of the 21st century, carrying profound implications for healthcare systems and social organization [1,2]. This process is typically characterized by a reduction in functional fitness, which increases the likelihood of adverse events, such as falls and mobility loss [3]. In this context, promoting active and healthy aging, which prioritizes the maintenance of functionality and quality of life, becomes a public health priority [4]. Within this demographic scenario, frailty and functional independence, frequently associated with autonomy, emerge as central indicators to characterize the aging process.

Frailty is defined as a clinical syndrome resulting from the cumulative decline of physiological systems, leading to greater vulnerability to adverse events [5]. The aging process is associated with progressive changes in the musculoskeletal, cardiovascular, immunological, and neurological systems, contributing to the loss of strength, endurance, and balance [3]. The presence of frailty is therefore associated with severe consequences, namely a higher risk of falls, disability, hospitalization, institutionalization, and mortality [3,6]. Often characterized by a physical phenotype, a combination of involuntary weight loss, fatigue, weakness, slowness, and reduced physical activity, frailty can act as a determining factor in the progressive loss of functional independence, directly impacting the quality of life of older adults [7]. However, beyond this purely physical model, more recent approaches consider frailty as a biopsychosocial construct that integrates cognitive, emotional, and social components [8,9]. This broader perspective has gained relevance, as factors such as social isolation, loneliness, or the lived experience of vulnerability can exacerbate the decline of older adults [10,11].

Studies show that the prevalence of frailty increases progressively with age, being particularly high in individuals aged 80 years or older [12]. Population-based studies indicate that advancing age is one of the main determinants of the transition from robust states to pre-frailty and frailty, reinforcing this phenomenon as a dynamic process related to the life course [13]. Recent literature also highlights significant differences between sexes. Consistently, women show a higher prevalence of frailty and pre-frailty compared to men of the same age group [10,14]. This phenomenon is described as the “health-survival paradox,” wherein women live longer but experience higher levels of frailty and functional disability. Explanations for these differences include biological factors, such as lower muscle mass and hormonal changes, as well as psychosocial and economic factors, such as greater exposure to poverty and social isolation throughout the life cycle [14]. On the other hand, despite a lower prevalence, frail men tend to present a higher risk of mortality, suggesting distinct aging trajectories between the sexes [15].

In contrast, autonomy is understood as the capacity to make decisions and manage one’s own life [4,16,17]. Functional decline results from multiple factors, including physiological changes, the presence of chronic diseases, frailty, and unfavorable environmental conditions [18]. The loss of autonomy has a direct impact on self-esteem and social participation and is often associated with increased dependence [19]. However, autonomy does not necessarily disappear with age or physical loss. It can transform from an autonomy based on independent execution to a supported or relational autonomy [20]. In this sense, the ability to make decisions and exert control over one’s own life can be maintained, even in the presence of functional limitations, provided that the environment respects the older adult’s personal preferences [2]. The preservation of autonomy thus depends not only on physical and mental health but also on the characteristics of the context. Facilitating, accessible, and safe environments can delay functional decline and improve quality of life [21]. Studies show that factors such as social support and a suitable physical environment, whether in the community or in nursing homes, can mitigate the impact of chronological age on the loss of autonomy [22].

### 1.1. The Impact of the Place of Residence on Aging

The place of residence can play a determining role in the well-being and health trajectory of older adults, acting not only as a physical setting but also as an active social and relational context. The concept of Aging in Place, that is, growing older in one’s own home and community, is valued in the literature and in public policies for promoting the continuity of routines, the preservation of personal identity, and a sense of belonging [23]. Remaining in the community is frequently associated with the maintenance of neighborhood networks and a greater sense of control over daily life. On the other hand, living at home can present significant challenges, especially when severe physical limitations or architectural barriers arise, or when the informal support network is insufficient to meet healthcare needs [24].

Nursing homes (or residential care facilities for older adults) emerge as a necessary response for individuals with a higher degree of dependence, offering continuous support, supervision, and specialized care. Studies indicate that these environments can improve safety and ensure immediate access to healthcare, but they also entail risks to individual autonomy [25]. The literature suggests that institutionalization can inadvertently reduce decision-making opportunities and promote sedentary behaviors, negatively affecting functional independence [19,26].

Regarding frailty, evidence indicates that levels tend to be substantially higher among institutionalized older adults compared to community-dwelling residents [27]. This discrepancy is partly explained by the admission criteria to the institutions themselves, which often presuppose an existing functional or cognitive decline. However, environmental factors within the institution, such as the lower motor demands of daily routines, can accelerate muscle mass loss and physical decline, exacerbating the frailty syndrome [24].

Concerning autonomy, the effects of the institutional environment are complex and vary according to the prevailing care philosophy. While in the community, autonomy is often exercised through the direct execution of tasks, in an institution, it can transform into delegated autonomy. However, excessively protective or rigid environments can compromise self-determination, leading to what the literature designates as learned dependence [25].

The relationship between these variables may be mediated by age. Advancing chronological age is known to be the main risk factor for frailty in both contexts [12]. However, the interaction between advanced age and institutionalization appears to have a cumulative effect on the loss of functionality. Recent studies suggest that the “oldest-old” in institutional settings show a faster decline in strength and balance than their peers in the community, reinforcing the need for motor intervention programs tailored to this specific context [3,19].

Despite the relevance of these dynamics, there is a scarcity of studies directly comparing populations residing in the community and in nursing homes in the Leiria region using validated instruments for the Portuguese population. Comparing these contexts is essential to understand how environmental, social, and organizational factors influence the aging process, allowing for the design of strategies that minimize the negative impact of institutionalization on frailty and autonomy.

### 1.2. Objectives of the Study

The primary objective of this study was to compare the levels of frailty and functional independence in older adults according to their place of residence. As secondary objectives, it aimed to analyze the association between age, frailty, and functional independence, considering the variables of place of residence and sex.

Based on the literature review, the following hypotheses were formulated: (a) community-dwelling older adults present lower levels of frailty and higher indices of autonomy compared to institutionalized older adults; (b) there is a positive correlation between age and frailty, and a negative correlation between age and autonomy, in both places of residence, being more pronounced in institutionalized older adults; and (c) there is a positive correlation between age and frailty, and a negative correlation between age and autonomy, in both sexes, being more pronounced in female older adults.

## 2. Materials and Methods

### 2.1. Study Design and Participants

This study followed a cross-sectional, observational, and analytical design. A convenience sample was used, including both community-dwelling and institutionalized older adults.

The sample size was calculated a priori using G*Power 3.1 software (Heinrich Heine Universitat, Dusseldorf, Germany) [28], considering the following parameters: an effect size of 0.5, a significance level (α) of 0.05, and a statistical power (1 − β) of 0.90. The calculation indicated the need to include at least 70 participants per group to ensure adequate statistical power. Regarding inclusion criteria, eligible participants were older adults aged 65 years or older with preserved verbal communication capacity. Additionally, sufficient visual and auditory acuity was required to understand the instructions and complete the instruments, alongside voluntary acceptance to participate and the ability to sign the informed consent form.

### 2.2. Data Collection Procedures

Before starting data collection, this study obtained a favorable opinion from the Ethics Committee of the Polytechnic Institute of Leiria (protocol code CE/IPLEIRIA/02/2026, dated 19 January 2026). The data collection procedure was structured into four main phases. In the first phase, contact was established with the management of Santa Casa da Misericórdia de Leiria and the HP Fitness gym to present the master’s project and the study’s objectives. In the second phase, following institutional authorization, clinical and technical teams were contacted to request support in collecting sociodemographic data. Additionally, their collaboration was requested to identify and recruit potential participants who met the inclusion criteria. In the third phase, the study was presented directly to potential participants. During this stage, all necessary clarifications were provided, and informed consents were collected, assuring the older adults of the voluntary nature of their participation and their freedom to withdraw at any time without any negative repercussions. In the final phase, the actual data collection took place, which included sociodemographic characterization and the administration of the instruments.

It should be noted that all ethical and data protection procedures, established by Article 21 of Law No. 58/2019 of the Assembly of the Republic of Portugal, were strictly followed. The collected data were used exclusively within the scope of this research and destroyed after the academic objectives were met.

### 2.3. Instruments

To collect data for the sociodemographic characterization of the sample, a customized questionnaire was used, which included the following variables: age, sex, marital status, educational level, and place of residence (community or institutionalized).

To assess frailty, the Portuguese version of the Tilburg Frailty Indicator, validated by Coelho et al. [29], was used. This instrument adopts a multidimensional approach to frailty and consists of 15 items divided into three domains: physical (8 items), psychological (4 items), and social (3 items). The total score ranges from 0 to 15. In this instrument, higher scores correspond to a higher level of frailty in the participant.

The level of functional independence in activities of daily living was assessed using the Barthel Index, applying the version validated for the Portuguese institutionalized population by Araújo et al. [30] and the Portuguese version by Junior et al. [31] for the community-dwelling population. This scale consists of 10 items that evaluate the individual’s ability to perform basic tasks such as feeding, personal hygiene, toilet use, transfers, and mobility. The global score ranges from 0 to 100 points. Unlike the previous instrument, a higher score on the Barthel Index (closer to 100) indicates greater functional independence, while lower values signal an increasing degree of dependence.

### 2.4. Statistical Analysis

Data processing and analysis were performed using IBM SPSS Statistics software, version 30.0 (IBM Corp., Armonk, NY, USA). Initially, a descriptive analysis was conducted for a detailed characterization of the sample. For categorical variables, absolute frequencies (*n*) and relative frequencies (%) were calculated. For continuous variables, the analysis included the calculation of measures of central tendency and dispersion. Prior to inferential analysis, the assumptions of normality and homogeneity of variances were assessed. The Shapiro–Wilk test indicated that the frailty scores followed a normal distribution in both the community (*p* = 0.243) and institutionalized (*p* = 0.068) groups. While the autonomy scores violated the normality assumption (*p* < 0.001), the parametric independent samples Student’s *t*-test was maintained. This decision is supported by the Central Limit Theorem and the robustness of the *t*-test for sample sizes greater than 30 participants per group. Homogeneity of variances was verified using Levene’s test. Subsequently, to test for statistically significant differences between groups, the independent samples Student’s *t*-test was used, with the significance level set at *p* < 0.05. In addition to statistical significance, the effect size was calculated to quantify the magnitude of the observed differences. For the *t*-test, Cohen’s d was used and interpreted according to reference values: 0.20 (small effect), 0.50 (medium effect), and 0.80 (large effect). To address the objective of analyzing the association between age, frailty, and autonomy based on the place of residence and sex, Pearson’s correlation coefficient was used, with the significance level set at *p* < 0.05. Finally, to control the confounding effect of age between the two places of residence, an Analysis of Covariance was conducted, setting age as a covariate.

## 3. Results

The total sample of the present study consisted of 77 participants, with a mean age of 80.16 years (SD = 8.68). A predominance of females was observed, representing 72.7% (*n* = 56) of the sample, compared to 27.3% (*n* = 21) for males. Regarding marital status, the majority of participants were either married or widowed, with both categories representing 45.5% of the sample each (*n* = 35). Regarding educational level, the sample was characterized by a low level of schooling, with the 4th grade (primary education) being the most frequent qualification (59.7%).

When analyzed according to the place of residence, the sample was evenly distributed, with 49.4% (*n* = 38) of the older adults living in the community and 50.6% (*n* = 39) institutionalized. It is important to highlight that the institutionalized participants presented a higher mean age compared to the community-dwelling residents. Differences were also observed in the distribution of marital status between the groups. While married participants prevailed in the community (60.5%), a higher prevalence of widowhood was observed in the institutions (59.0%). The detailed characterization of the sociodemographic variables for the total sample and by place of residence is described in Table 1.

The obtained results, presented in Table 2, revealed statistically significant differences regarding autonomy. Community-dwelling participants showed significantly higher levels of independence compared to institutionalized older adults (*p* < 0.001), registering a large effect size (*d* = 1.03). However, contrary to speculation, no statistically significant differences were found in frailty levels between the two groups (*p* = 0.674). Although the mean frailty was slightly higher in institutionalized older adults compared to those in the community, this difference has no significant relevance.

After adjusting for age, the differences in autonomy levels between community-dwelling and institutionalized older adults remained statistically significant (F(1, 73) = 15.038, *p* < 0.001, partial eta squared = 0.171). Furthermore, age was not a significant covariate in this model (F(1, 73) = 0.156, *p* = 0.694, partial eta squared = 0.002), confirming that the place of residence is an independent determinant of functional autonomy, regardless of the age discrepancy between groups.

In community-dwelling older adults, a moderate negative correlation was found between frailty and functional independence (*r* = −0.495; *p* = 0.002). This indicates that higher levels of frailty are significantly associated with lower autonomy. Regarding age, no statistically significant correlations were found with the other variables, although the association with frailty was at the threshold of significance (*p* = 0.052). In the group of institutionalized older adults, a similar pattern was observed, highlighting a strong and significant negative correlation between frailty and functional independence (*r* = −0.641; *p* < 0.001). In this group, age showed no significant association with either frailty or functional independence. For more information, see Table 3.

In males, the results evidenced a strong negative correlation between frailty and functional independence (*p* < 0.001). Additionally, a moderate negative correlation was verified between age and functional independence (*p* = 0.050), suggesting that, in this group, advancing age tends to be associated with a decrease in independence. Regarding females, the inverse association between frailty and autonomy was also confirmed (*r* = −0.459; *p* < 0.001). However, unlike the male group, no significant correlations were found between age and the other studied variables.

## 4. Discussion

The present study sought to analyze the dynamics between the place of residence, frailty, and functional independence in a sample of Portuguese older adults. The collected evidence suggests that, although the residential context (community vs. institution) is a discriminating factor in autonomy levels, its relationship with frailty levels appears more complex than what the literature frequently postulates.

Regarding autonomy, the results corroborated the initial hypothesis, showing that community-dwelling older adults possess significantly higher levels of autonomy (*p* < 0.001) with a large effect size (*d* = 1.03) compared to institutionalized individuals. This finding aligns with the perspective of Aging in Place advocated by Wiles et al. [23] and Sixsmith and Sixsmith [25], who suggest that the domestic environment imposes a continuous functional demand, from household management to neighborhood mobility, acting as a constant motor and cognitive stimulus. In contrast, the organizational structure of institutions, while ensuring safety and care, tends to replace the execution of basic tasks such as feeding, hygiene, and transfers. This precipitates what several authors identify as environmentally accelerated functional decline or learned dependence [19,26]. The lower score on the autonomy index in the institutionalized group, therefore, reflects not only the biological condition that motivated the admission [27] but potentially the hypokinesia associated with institutional routines [4]. On the other hand, no statistically significant differences were found in frailty levels between the two groups (*p* = 0.674). Therefore, the hypothesis that institutionalized older adults would present higher perceived frailty was not supported in this specific sample.

Regarding the correlational analysis, the data confirmed the hypothesis that frailty and autonomy are inversely associated in both residential contexts. However, the magnitude of this association proved to be notably higher in the institutionalized group (*r* = −0.641; *p* < 0.001) compared to community residents (*r* = −0.495; *p* = 0.002). These results suggest that, although frailty is inversely associated with autonomy, its impact on the loss of autonomy is exacerbated by the institutional context. It is possible to speculate that, in a community setting, older adults manage to implement compensatory strategies, such as the use of technical aids or adapting the pace of tasks, that allow them to maintain autonomy even in the presence of mild to moderate frailty, which is consistent with the perspective of Aging in Place and environmental adaptation [23]. Conversely, in residential care facilities for older adults, the rigidity of organizational routines and the substitution of self-care with service provision can cause small increments in frailty to result in disproportionate dependence, validating the deficit accumulation theory proposed by Rockwood and Mitnitski [11]. In this scenario, the margin for autonomy is smaller, as any additional frailty, whether physical, psychological, or social, can translate into a loss of functional independence, a phenomenon described in the literature as a negative interaction between individual vulnerability and environmental pressure [17,26].

Concerning the age variable, the results revealed a complex and distinct dynamic between sexes. Contrary to the hypothesis of a universal linear association, chronological age showed no significant correlation with frailty in any of the analyzed subgroups. This finding, at first glance counterintuitive given the biological process of senescence [1,2], can be interpreted in light of the survival bias phenomenon in older age samples. As indicated in the literature [3,13], cohorts of older adults at advanced ages (i.e., in this sample with a mean of 80.16 years) represent a selected group of survivors who have possibly developed adaptive mechanisms that dissociate chronological age from the accumulation of frailty.

However, when analyzing autonomy, a specificity was found in males. It was the only group where advancing age was significantly associated with a decrease in functional independence (*r* = −0.43; *p* = 0.050). In women, age was not a predictor of autonomy. This suggests that functional decline in men may follow a more linear trajectory dependent on chronological age, whereas in women, this decline appears to be governed by factors other than just years of life, reinforcing the heterogeneity of female aging. This disparity introduces the discussion about a “health-survival” paradox [10,14], which is a robust demographic and epidemiological phenomenon describing the discrepancy between life expectancy and health expectancy based on sex. These data seem to indicate that women live longer than men but may experience worse health conditions, more disability, and a higher prevalence of frailty during those additional years.

The data in Table 4 show that the association between frailty and autonomy is stronger in men (*r* = −0.72; *p* < 0.001) than in women (*r* = −0.45; *p* < 0.001). The interpretation of these coefficients suggests that men have a lower functional tolerance to frailty. Conversely, women, although frequently presenting a higher prevalence of comorbidities [14], seem able to maintain levels of relative autonomy even in the presence of frailty. This evidence aligns with the perspective of Kojima et al. [15], who indicate that, although women live longer with frailty, frail men have a higher risk of mortality and functional collapse. Thus, the results of this study reinforce the need for a differentiated clinical approach based on sex; namely, in older men, vigilance must be doubled, as the intersection between advanced age and frailty appears to lead to a more abrupt loss of autonomy than in females.

Finally, it is important to reflect on whether frailty and functional autonomy, as operationalized herein, fully capture the multidimensional nature of vulnerability across care settings. Emerging research highlights that integrating electronic health records and diagnostic patterns can provide an additional, highly effective lens to identify and prioritize risk trajectories. Some clinical profiles derived from electronic health records carry stronger predictive signals for adverse outcomes than self-reported measures alone [32]. Future work should integrate these complementary indicators to improve vulnerability assessment in gerontology.

### Limitations of the Study

The interpretation of the results of the present study must be considered in light of a set of limitations which, although inherent to the exploratory nature of the research, influence the generalization and depth of the conclusions. The main limitation lies in the cross-sectional design adopted, which restricts the analysis to a single point in time, preventing the establishment of causality between the place of residence and the dependent variables. Consequently, the direction of the observed associations remains speculative. That is, it is not possible to unequivocally determine whether the lower autonomy recorded in institutionalized older adults is a direct effect of the hypokinesia and learned dependence promoted by the institutional environment, or if, conversely, it was the prior functional decline that precipitated the need for institutionalization, as suggested in the predictive models of Luppa et al. [27].

Additionally, the convenience sampling technique and the sample size proved to be limited when stratifying by subgroups. The small number of male participants may have compromised the statistical power to detect interactions between sex, frailty, and autonomy. It is equally plausible to consider the existence of a strong selection bias in the community group. Since recruitment was partly conducted through a fitness center, it likely attracted healthier, more active, and socially integrated individuals. This phenomenon may have artificially amplified the observed difference in autonomy compared to the institutionalized group, masking the reality of older adults in the community with greater isolation and undiagnosed dependence. Furthermore, post hoc power analysis revealed that our total sample size of 77 participants achieved a statistical power of approximately 58% to detect a medium effect size (d = 0.50). This indicates that the study is underpowered compared to the ideal threshold calculated a priori, warranting caution when interpreting the results.

A critical limitation for interpreting frailty levels relates to the exclusive self-report nature of the instruments used. Although validated, the Tilburg Frailty Indicator and Barthel Index measure the perception of frailty and autonomy, rather than objective physical capacity. The absence of differences in frailty between settings might reflect measurement artifacts, response adaptation, or contextual reporting bias rather than true clinical equivalence. The lack of data triangulation with objective physical performance tests, such as gait speed or handgrip strength, prevented the distinction between perceived and physical frailty phenotypes. Furthermore, this study did not control relevant confounding variables, such as cognitive status, polypharmacy, or history of falls, which could have offered a more granular understanding of the mechanisms underlying autonomy loss.

Despite these limitations, the obtained results open relevant doors for scientific development in the field of gerontomotricity. Future investigations should prioritize longitudinal designs that track the trajectory of frailty during the transition from the community to the institution, allowing the institutional effect to be isolated from natural biological decline. It would also be pertinent to adopt a mixed assessment approach, combining self-report scales with aging biomarkers and functional fitness tests, to discern more accurately whether the reported frailty reflects a psychological experience or a physiological failure. Finally, given the confirmation of the health-survival paradox, it is suggested that future studies qualitatively explore the coping strategies that allow women to maintain functionality despite high frailty, contrasting with the abrupt functional breakdown observed in men, in order to design differentiated and more effective interventions for promoting active aging.

## 5. Conclusions

The present study contributes to the literature by demonstrating that the place of residence is a discriminating factor for autonomy, but not necessarily for frailty levels when evaluated from a multidimensional perspective. It is concluded that institutionalized older adults present a significantly higher functional vulnerability, which reinforces the urgent need to reconfigure care models in long-term care facilities. It seems imperative to transition from an assistentialist paradigm (i.e., “doing for the older adult”) to a rehabilitative and autonomy-promoting paradigm (i.e., “doing with the older adult”), integrating multicomponent exercise programs and cognitive stimulation into daily routines.

The absence of differences in frailty between contexts leads to the existence of an invisible community-dwelling population, whose biopsychosocial needs may be neglected until the acute moment of institutionalization. Future research should prioritize longitudinal designs that track the trajectory of frailty during the transition from the community to the institution, incorporating objective markers of functional fitness and controlling for the impact of biopsychosocial variables, in order to better design active and healthy aging policies.

## Figures and Tables

**Table 1:** Sociodemographic characterization of the sample and subsamples.

Variables	Total (N = 77)	Community (N = 38)	Institution (N = 39)
Age (years)			
M ± SD	80.16 ± 8.68	76.55 ± 7.47	83.67 ± 8.42
Sex, *n* (%)			
Female	56 (72.7%)	28 (73.7%)	28 (71.8%)
Male	21 (27.3%)	10 (26.3%)	11 (28.2%)
Marital Status, *n* (%)			
Single	2 (2.6%)	0 (0.0%)	2 (5.1%)
Married	35 (45.5%)	23 (60.5%)	12 (30.8%)
Divorced	5 (6.5%)	3 (7.9%)	2 (5.1%)
Widowed	35 (45.5%)	12 (31.6%)	23 (59.0%)
Educational Level, *n* (%)			
No formal education	2 (2.6%)	1 (2.6%)	1 (2.6%)
1st Cycle (up to 4th grade)	50 (64.9%)	24 (63.2%)	26 (66.7%)
2nd Cycle (5th to 6th grade)	7 (9.1%)	3 (7.9%)	4 (10.3%)
3rd Cycle (7th to 9th grade)	7 (9.1%)	3 (7.9%)	4 (10.3%)
Secondary Education (12th grade)	3 (3.9%)	1 (2.6%)	2 (5.1%)
Higher Education	8 (10.4%)	6 (15.8%)	2 (5.1%)

Note: M = Mean; SD = Standard Deviation; *n* = absolute frequency; % = percentage.

**Table 2:** Comparison of frailty and autonomy according to the place of residence.

Variable	Community	Institution	*t*	*p*	Cohen’s d
	M ± SD	M ± SD			
Frailty	5.92 ± 2.49	6.15 ± 2.36	−0.422	0.674	−0.10
Autonomy	94.21 ± 10.81	75.53 ± 23.21	4.497	<0.001	1.03

Note: M = Mean; SD = Standard Deviation; *t* = *t*-test value; *p* = level of significance; *d* = Cohen’s effect size.

**Table 3:** Correlation matrix of the variables according to the place of residence.

Place of Residence	Variables	1	2	3
Community	1. Age	-	−0.31	−0.04
	2. Frailty		-	−0.49 *
	3. Autonomy			-
Institution	1. Age	-	−0.22	−0.05
	2. Frailty		-	−0.64 **
	3. Autonomy			-

Note: * *p* < 0.01; ** *p* < 0.001.

**Table 4:** Correlation matrix of the variables according to sex.

Sex	Variables	1	2	3
Male (*n* = 21)	1. Age	-	−0.27	−0.43 *
	2. Frailty		-	−0.72 **
	3. Autonomy			-
Female (*n* = 56)	1. Age	-	−0.21	−0.21
	2. Frailty		-	−0.45 **
	3. Autonomy			-

Note: * *p* < 0.01; ** *p* < 0.001.

## Data Availability

The data presented in this study is available on request from the corresponding author. The data is not publicly available due to privacy and ethical restrictions regarding participant anonymity.
